# The rise of methods-driven science: How Methods Papers Reshaped the Research Landscape

**DOI:** 10.1038/s44319-026-00873-7

**Published:** 2026-07-21

**Authors:** Alexander Krauss, Ariel Rosenfeld, Lutz Bornmann

**Affiliations:** 1 Spanish National Research Council, Barcelona, Spain; 2 London School of Economics, London, UK; 3 Department of Information Science and Applied Artificial Intelligence, Bar-Ilan University, Ramat-Gan, Israel; 4 Science Policy and Strategy Department, Max Planck Society, Munich, Germany

**Keywords:** History & Philosophy of Science, Methods & Resources, Science Policy & Publishing

## Abstract

While scientific progress is often attributed to theoretical and empirical advances, the underlying methodological innovations that enable these advances are frequently overlooked. We found that the share of methods papers doubled from 20% in 1980 to 40% in 2019 across all natural sciences, indicating a general transition toward method- and technique-driven science.

**Figure:**
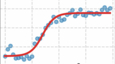

The progress of science is often narrated as a series of theoretical shifts and empirical advances. Yet, theoretical and experimental breakthroughs rest on a frequently underappreciated basis: the methodologies that make them possible. From gene amplification and DNA sequencing to modelling complex systems and analysing molecular structures, methods have been defining the limits of scientific observation and experimentation. One author (AK) has already highlighted the importance of methods for research in a series of publications, such as (Krauss, 2026).

Based on citation metrics, the most impactful publications in modern science are increasingly not primary theoretical or experimental findings, but the methodological innovations that enable and support them (Pearson et al, 2025; van Noorden et al, 2014). For instance, PCR (Saiki et al, 1985), DNA sequencing (Sanger et al, 1977) and the Lowry assay for protein analysis (Lowry et al, 1951) all revolutionised biology by paving the way for countless subsequent studies. Ultimately, these iconic ‘super-cited’ methods papers—which introduce a new procedure, algorithm, tool, instrument or technique—frequently overshadow the very scientific breakthroughs they facilitate.

“… the most impactful publications in modern science are increasingly not primary theoretical or experimental findings, but the methodological innovations that enable and support them.”

## The role of methods papers

Do these high-impact methods papers represent isolated anomalies, or do they indicate a broader structural shift across science? Put simply: is scientific research increasingly reliant on methods? To investigate this, we examined the role of methods papers across all levels of scientific impact (Krauss et al, 2026). We assembled a stratified corpus of journal articles indexed in the Web of Science (WoS, Clarivate) from 1980 to 2019. The data were annually stratified into five specific citation impact tiers (Bornmann and Williams, 2020): the top 1%, 25% (24.5–25.5%), median (49.5–50.5%), 75% (74.5–75.5%) and bottom 1%. Our analysis was restricted to disciplines with established empirical traditions—namely the natural sciences, medical and health sciences, engineering and technology, and agricultural sciences—yielding a dataset of 3,117,335 papers. To verify the robustness of our results, we compiled a parallel validation dataset from the Scopus database (Elsevier). WoS served as our primary source because of its historical depth, which is crucial for identifying potential structural transformations starting in the 1980s. Scopus provided extensive coverage for validation from the mid-1990s onwards (Krauss et al, 2026).

To identify methods papers, we employed a supervised machine-learning approach. We built a balanced ground-truth dataset by manually selecting ten pairs of journals, 20 in total. Each pair matched a specifically ‘methods-oriented’ journal, such as *Nature Methods* or *Journal of Neuroscience Methods*, with a ‘general’ journal of comparable scope and reputation. This paired structure enabled the classifier to learn the distinct lexical features of methods papers rather than relying on disciplinary cues or citation metrics. For all 180,454 articles published in these journals during our study period, we extracted the titles and abstracts, vectorized the text via standard term frequency-inverse document frequency (TF-IDF), followed by training a logistic regression classifier (Manning et al, 2008). We evaluated this model against modern alternatives, including SciBERT (Beltagy et al, 2019) and large language models such as GPT-4. Ultimately, the TF-IDF approach delivered the best performance (accuracy: 93.9%; F1:0.899) while preserving both high interpretability and reproducibility. Finally, we deployed the trained classifier across the entire WoS dataset, where it assigned a probability score to each paper. Any publication with a probability score above 0.5 was categorised as a ‘methods paper.’

## A fundamental element of research

**Figure 1:**
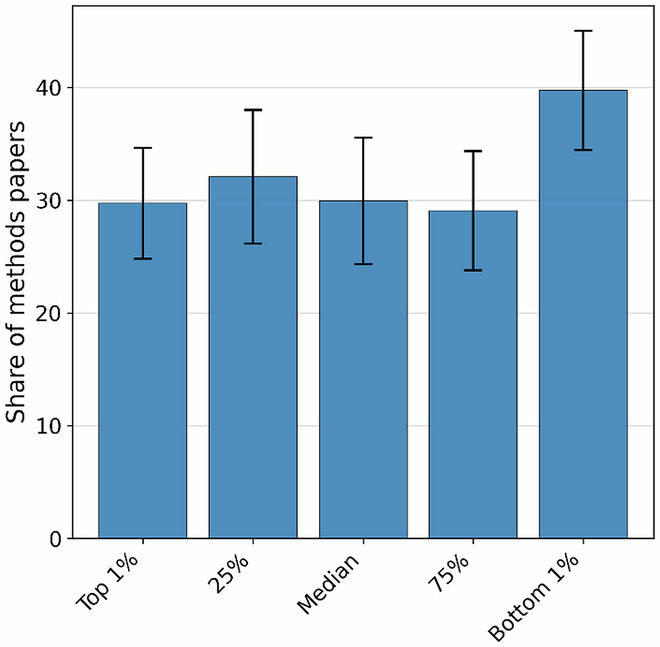
Share of methods papers across five stratified citation levels, ranging from the top 1% (highest citation impact, normalised by discipline and year) to the bottom 1% (lowest).

The results show that methods papers are not restricted to the upper echelons of scientific papers; rather, they are prevalent throughout the entire literature (Fig. 1). At every citation tier, methods papers consistently make up between 30 and 40% of all publications—from the top 1% of highly cited articles to the broader foundation of everyday scientific work. This finding challenges the assumption that methods papers only matter when they achieve ‘citation-classic’ status. Instead, they constitute a fundamental element of research output regardless of their overall impact.

“At every citation tier, methods papers consistently make up between 30% and 40% of all publications—from the top 1% of highly cited articles to the broader foundation of everyday scientific work.”

**Figure 2:**
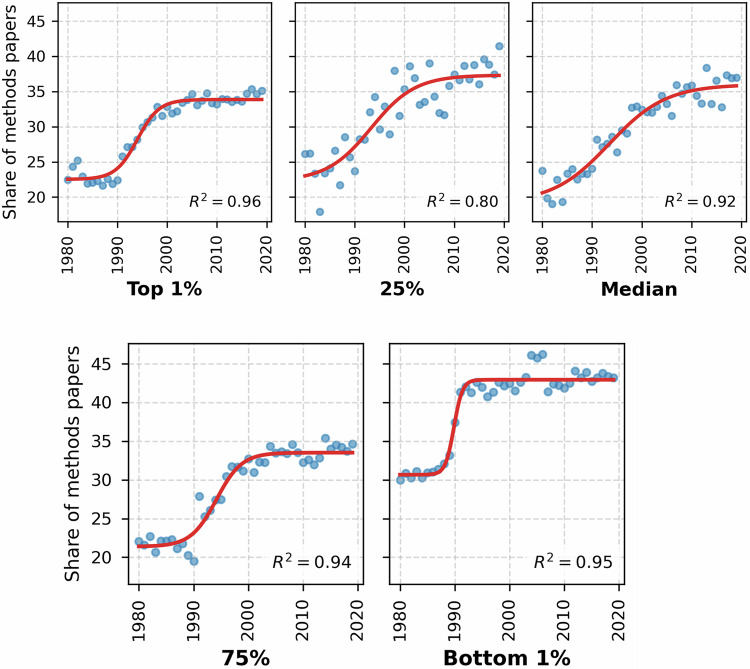
Prevalence of methods papers by citation level across all disciplines, over time.

Our findings thereby indicate a profound structural shift in the production of scientific knowledge over time. From 1980 to 2019, the proportion of methods papers doubled, expanding from roughly 20 to 40% across the literature (Fig. 2). This increase exhibits a classic S-shaped curve, which indicates a systemic transformation rather than a slow evolution. Within the top 1% of highly cited papers, the share of methods had grown at a modest rate of 0.11 percentage points annually until the late 1980s. Between 1988 and 1991, this growth rate accelerated more than 30-fold, reaching 3.42 percentage points per year. After this inflection point, the upward trend persisted, suggesting that this methodological shift is a permanent characteristic of research rather than a temporary anomaly.

**Figure 3:**
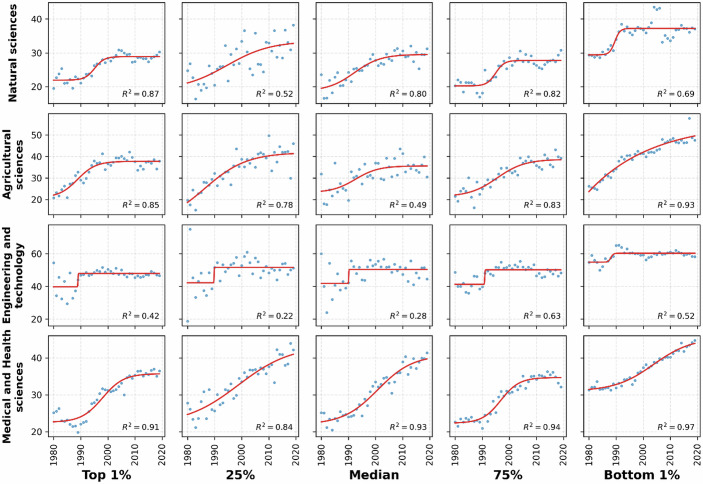
Share of methods papers across citation impact levels and disciplines, over time.

While the upward trend is omnipresent and the sigmoid temporal pattern is common across disciplines, the timing and intensity of the acceleration differ slightly by discipline (Fig. 3). The natural sciences display an early onset—accelerating in the late 1980s—whereas the medical and health sciences show a delayed onset with a less pronounced inflection point. Take, for example, the innovative technique called optical tweezers—like tweezers, this laser-based tool grabs particles using beams of light, enabling biologists since 1987 to manipulate DNA strands, viruses and even living cells with great precision. Optical tweezers are used, for instance, to study the structure of proteins or run diagnostic tests for malaria. Or take the discovery of cyclin in 1982: the protein that controls the cell cycle. It was enabled by the invention of the slab SDS polyacrylamide gel (electrophoresis)—a crucial method for biomedical research. Another example is the Human Genome Project, launched in 1990 using novel tools such as automated DNA sequencing and sequence-tagged site maps (International Human Genome Sequencing Consortium, 2004). The agricultural sciences follow the natural sciences and medical and health sciences with a similarly strong upward trend in the 1990s, while engineering and technology exhibited earlier growth in the late 1980s. Despite these variations, the overarching pattern remains remarkably consistent: science as a whole—across disciplines, citation levels and decades—has decisively transitioned toward methods papers.

“… the overarching pattern remains remarkably consistent: science as a whole—across disciplines, citation levels and decades—has decisively transitioned toward methods papers.”

## The forces behind methods

To explain this methodological surge, we identified three interrelated structural forces.

### First, the computational and digital revolution

Since the early 1990s, scientific research has become fundamentally more computational, driven by the rapid spread of affordable personal computers, early internet infrastructure and software development (Hilbert and López, 2011). These technologies facilitated digital imaging, advanced statistical techniques, machine-learning methods, algorithmic computations and high-throughput experimentation, and enabled an unprecedented level of integration between physical instruments and computational tools—essentially, instrument-software integration at scale. Computer science helped spearhead this early transition by developing general-purpose tools that were subsequently adopted across other disciplines. The PCR method (Saiki et al, 1985) and the Human Genome Project acted as major catalysts in the biomedical sciences. They accelerated the development of new automated instruments, sequencing platforms and statistical genetics, driving methodological advances that quickly diffused throughout biology and medicine (International Human Genome Sequencing Consortium, 2004). Ultimately, the pairing of affordable computing power with sophisticated software has transformed experimental design, data collection and analysis across all scientific disciplines (Krauss, 2025).

### Second, the rise of data-intensive science

The shift toward data-driven science—often characterised as the era of ‘big data’—significantly increased the demand for general-purpose methods and tools. From genomics to astronomy, the influx of big data expanded the need for systems capable of acquiring, processing, cleaning, analysing, storing and visualising massive volumes of information (Bell et al, 2009; Hey et al, 2009). Furthermore, the proliferation of statistical programs and computational libraries (such as Python, R and MATLAB) accelerated methodological innovation and lowered the barrier to implementing complex techniques, especially since platforms such as R are freely available.

“The shift toward data-driven science—often characterised as the era of ‘big data’—significantly increased the demand for general-purpose methods and tools.”

### Third, expanding interdisciplinarity

Research has increasingly integrated and recombined methodological approaches and techniques from various fields, favouring portable methods that can be widely reused over disciplines (Larivière et al, 2015). Because these reusable methods cater to broader communities, they naturally tend to achieve higher levels of citation and widespread adoption.

The structural break we identified in the 1990s coincides with several mutually reinforcing technological and institutional transitions. By the early 1990s, personal computers from companies like IBM and Apple had become widely affordable for research laboratories (Ceruzzi, 2003). Introduced in 1991, the World Wide Web facilitated an unprecedented level of sharing for data, code and methodological protocols (Berners-Lee and Fischetti, 1999). Software packages such as SPSS, Stata, and R became increasingly accessible to non-specialists, making advanced statistical methods available to broader scientific communities (Fox, 2008). The digitisation of laboratory equipment drove the development of digital microscopy, sequencing, detectors and imaging, alongside other computer interfaces and automated data acquisition systems (Krauss, 2025). Large-scale initiatives like the Human Genome Project acted as global accelerators. By driving the demand for high-throughput sequencing and bioinformatics tools, they fostered innovations that subsequently spread throughout biology and medicine.

The synergistic integration of computing, statistics and digital instrumentation has exponentially increased scientific efficiency, driving a rapid acceleration of methodological innovation that was especially pronounced in the early 1990s. From biology and medicine to physics, computational breakthroughs have directly paved the way for novel instruments, analytical techniques and experimental setups, thereby significantly enhancing scientific productivity (Hey et al, 2009).

While our results show a consistent upward trend in methods papers across disciplines, the exact timing varies due to differences in adoption. In physics and chemistry, the early growth of methods papers aligns with the spread of high-performance computing, simulation-based inference, instrument automation, and advanced spectroscopy and imaging tools, which have collectively enabled greater experimental advances. In biomedicine, the steepest surge corresponds to the rapid diffusion of high-throughput technologies—including PCR, automated DNA sequencing, fluorescence and digital microscopy, alongside other imaging technologies and, later, microarrays. These innovations reshaped experimental workflows and created a sustained demand for reusable methods. In engineering and computer science, the rise began in the late 1980s, reflecting the expansion of algorithmic tools, numerical optimisation methods and computational architectures that laid the foundation for modern machine-learning and software-driven tool development. These advancements, in turn, helped spur growth in other disciplines. In agriculture and the environmental sciences, we observe a rise, primarily in the 1990s and 2000s, which is consistent with the adoption of digital sensors, Geographic Information System (GIS) technologies, remote-sensing platforms and bioinformatics pipelines. Ultimately, the integration of satellite imagery, precision-agriculture tools, and environmental-modelling software helped drive the methodological transformation in these disciplines (Krauss, 2025).

## Incentives for method innovations

Our findings point to a need to rethink how we conceptualise and conduct science. We should recalibrate scientific evaluation systems so that method architects are properly credited for creating shared research infrastructure, incentivised to publish in leading journals, fairly assessed in tenure-track processes, and rewarded for innovations that enable downstream scientific advances (Krauss, 2026).

“We should recalibrate scientific evaluation systems so that method architects are properly credited…”

Beyond the immediate implications for funding, incentives and tenure, these results challenge the traditional model of science as a mere succession of theoretical paradigms and experimental findings. Instead, they highlight a system where progress is increasingly method-driven, and where the pace of discovery is set by the availability of novel methods. In other words, 'to make exciting advances, researchers rely on relatively unsung papers to describe experimental methods, databases and software' (van Noorden et al, 2014, p. 550). The ‘methodisation’ of science we uncover implies that the boundary between ‘core research’ and ‘technical support’ is rapidly dissolving. Science policy must therefore evolve to treat methods, software, databases and protocols not as secondary byproducts, but as primary scientific outputs that require sustained investment, distinct evaluation metrics, and dedicated professional pathways for the architects of scientific infrastructure. Ensuring that the impact of these architects is rewarded is essential for sustaining the entire scientific ecosystem.

“… these results challenge the traditional model of science as a mere succession of theoretical paradigms and experimental findings.”

Although our analysis comprehensively captures the digital transition up to 2019, it concludes at the threshold of the AI era—an interesting topic for future research. The widespread integration of large language models and AI agents introduces complex new dynamics into the scientific landscape. On one hand, their capacity to autonomously generate code and formulate experimental protocols could significantly lower the barriers to methodological innovation. On the other hand, the commoditization of scientific processes by AI could simply drive a massive expansion in general research output. Consequently, a critical question for the coming decade remains as to which force will ultimately dominate: will AI catalyse a radical ‘hyper-methodisation’ of science, or will it merely amplify the overall volume of published literature?

## Supplementary information

Peer Review File
