# Supplementary material for: The rise of methods-driven science: How Methods Papers Reshaped the Research Landscape
Source: EMBO Rep. 2026 Jul 21;27(15):4199–203. doi: 10.1038/s44319-026-00873-7 (PMC13457870; doi:10.1038/s44319-026-00873-7)
Supplement: Supplementary file 1 [file 44319_2026_873_MOESM1_ESM.pdf]

EMBO reports

The Rise of Methods-Driven Science: How Methods Papers Reshaped the Research Landscape

Alexander Krauss, Ariel Rosenfeld, and Lutz Bornmann

Corresponding author(s): Alexander Krauss (a.krauss@lse.ac.uk)

Review Timeline:

Submission Date:	6th Jul 26
Accepted:	7th Jul 26

Editor: Holger Breithaupt

Transaction Report: This article was editorially reviewed.
